# Long-term outcome and quality of life after CNS cavernoma resection: eloquent vs. non-eloquent areas

**DOI:** 10.1007/s10143-021-01572-8

**Published:** 2021-06-23

**Authors:** Loay Shoubash, Jörg Baldauf, Marc Matthes, Michael Kirsch, Matthias Rath, Ute Felbor, Henry W. S. Schroeder

**Affiliations:** 1 Department of Neurosurgery, University Medicine Greifswald, Ferdinand-Sauerbruch-Strasse, 17475 Greifswald, Germany; 2 Department of Neuroradiology, University Medicine Greifswald, Greifswald, Germany; 3 Department of Human Genetics, University Medicine Greifswald and Interfaculty Institute of Genetics and Functional Genomics, University of Greifswald, Greifswald, Germany

**Keywords:** Cavernoma, Cavernous malformation, Eloquent area, Quality of life, Surgical resection, Neurological outcome

## Abstract

The aim of this study is to analyze the long-term quality of life after surgery of cavernoma. A monocentric retrospective study was conducted on 69 patients with cavernoma treated microsurgically between 2000 and 2016. The eloquence was adopted from Spetzler-Martin definition. A most recent follow-up was elicited between 2017 and 2019, in which the quality of life (QoL) was evaluated with the Short Form-12 questionnaire (SF12). Forty-one lesions were in eloquent group (EG), 22 in non-eloquent group (NEG), 3 in orbit, and 3 in the spinal cord. Postoperative worsening of the modified Rankin scale (mRS) occurred in 19.5% of cases in EG versus 4.5% in NEG. After a mean follow-up of 6.5 years (SD 4.6), the neurological status was better or unchanged compared to baseline in 85.4% of EG and 100% of NEG. Regarding QoL assessment of 44 patients (EG *n* = 27, NEG *n* = 14) attended the last follow-up. Patients after eloquent cavernoma resection reported a non-inferior QoL in most SF12 domains (except for physical role) compared to NEG. However, they reported general health perception inferior to norms, which was affected by the limited physical and emotional roles. At a late follow-up, the surgical morbidity was transient in the NEG and mostly recovered in the EG. The QoL comparison between eloquent and non-eloquent cavernomas created interesting and new data after prolonged follow-up. These results add value for decision-making as well as patient counseling for future encountered cases. Preoperative evaluation of QoL is recommended for future studies to assess QoL dynamics.

## Introduction

Cavernoma or cavernous malformation (CM) is a benign non-shunting vascular malformation that is prone to bleed [25]. They account for 5–15% of the central nervous system (CNS) vascular malformations [10] and present with seizures, focal neurologic deficits (FND), or incidentally [19]. Disease prevalence ranges from 0.4 to 0.8% [4, 13, 23]. The overall annual bleeding risk is 2.4% patient/year [12]. In previously unruptured CM, the bleeding risk is 0.3–2.8% patient/year, but the risk reaches 6.3–32.2% patient/year once the cavernoma bled [2, 31].

In asymptomatic cavernomas, a “wait and see” conservative management could be the first choice [16, 20]. Previous publications reported, however, that a bad preoperative performance level at the presentation is a predictive factor of a poor outcome. That means, “wait and see” strategy for eloquent CM after the first hemorrhage will leave these patients at risk of neurological deterioration from substantial bleedings, therefore decreasing the chance for complete recovery after surgery [5, 6, 8]. Given the increased risk of rehemorrhage, microsurgical resection remains the definitive treatment for CNS cavernomas [11].

The main concern is the outcome of surgical treatment. There is a need to validate the quality of life of the patients after surgical resection of the cavernoma, especially in deep or eloquent areas. By reviewing the literature, we found many studies that assess the outcome after CNS cavernoma surgery and to a lesser extent after surgery of cavernomas located in eloquent regions [6, 14, 15, 24, 28, 30, 36, 39]. However studies that assess the QoL are still rare. Only four studies assess the quality of life of these patients, including three studies that evaluated brainstem cases only [7–9, 17].

We performed this study to investigate two main aspects: (1) the neurological outcome and (2) quality of life after CNS cavernoma resection.

## Methods

### Participants and study design

From January 2000 to December 2016, 74 patients with cavernoma treated surgically at the University Hospital Greifswald were evaluated in this retrospective clinical study. This study was approved by the Human Research Ethics committee of the University of Greifswald (Study ID: BB 031/18).

### Inclusion criteria and information gathered

The inclusion criteria were a histopathological confirmation of CNS cavernoma and complete data sets of the patients. Five patients were excluded from the study. Two had no clear histopathological confirmation of CM, and the remaining three patients were lost to follow-up, leaving a total of 69 subjects included in the study. Clinical charts, imaging studies, operation notes, and follow-up notes were reviewed.

Information regarding the patient’s gender, mean age at the surgery, location of the lesion, signs and symptoms, duration of the complaint, medications including antiepileptic drugs (AED) past medical history, preoperative MRI findings (including location and size [maximum diameter] of CMs and Zabramski classification [37] associated developmental venous anomaly (DVA)), length of stay, and length of the operation were gathered. Genetic counseling was offered to patients with a positive family history of CMs and multiple cavernomas.

The neurological examination (alertness/consciousness, orientation, dizziness, headache, cranial nerve status, sensomotoric deficit, gait, epilepsy) was standardized pre-and postoperatively according to the modified Rankin Scale (mRS) [22]. Based on the mRS, patients who presented with debilitating seizures or headaches were given mRS 1. In contrast, the presentation of simple non-disabling fit or chronic sporadic recurrent headaches that are controlled on medications considered mRS 0. Postoperative change of at least one grade of mRS was defined as better or worse. Table 1 shows the distribution of the patients according to mRS. A mRS score ≤ 2 was defined as a favorable outcome, where a mRS score > 2 was related to an unfavorable outcome.

**Table 1:** Modified Rankin Scale demonstrates the corresponding results from the study

mRS [22]	At presentation: EG	NEG	Directly postop: EG	NEG	Late follow-up: EG	NEG
0	6	7	13	11	21	14
1	23	14	16	9	9	7
2	4	0	5	1	6	0
3	3	0	4	0	1	0
4	5	1	2	1	1	0
5	0	0	1	0	3	0
6	0	0	0	0	0	1 +

*EG* eloquent group; *mRS* modified Rankin scale; *NEG* non-eloquent group: *postop* postoperatively

^#^ some patients had a transient FND, and they had mRS 0 at the presentation

+ died from another disease

### Eloquence

Eloquence was adopted from Spetzler-Martin definition [29]. The following CM locations were treated: sensorimotor *n* = 14, basal ganglia *n* = 2, language *n* = 8, visual pathway *n* = 5, deep cerebellar nuclei or cerebellar peduncles *n* = 4, and brainstem *n* = 8. Twenty-two cavernomas were found in non-eloquent areas. Three lesions were located in the orbit and 3 in the spinal cord. Eight patients had multiple cavernomas. Detailed localization is summarized in Table 2.

**Table 2:** Anatomical and functional location distribution of the surgically resected CM

Anatomical location distribution		Functional location distribution	
	No. of patients		No. of patients
Supratentorial	47	Eloquent [29]	41
Frontal	18	Sensomotoric region	14
Temporal	13	Language	8
Parietal	7	Visual	5
Parietooccipital	5	Basal ganglia	2
Occipital	2	Cerebellum deep nuclei, and peduncles	4
Basal ganglia	2	Brainstem	8
Cerebellar	8	Non-Eloquent	22
Brainstem	8	Frontal	7
Midbrain	1	Temporal	8
Pons	4	Parietal	3
Ponto-medullary	1	Cerebellar hemisphere	4
Medulla oblongata	2	occipital	0*

Others: spinal cord *n* = 3, orbital *n* = 3, multiple *n* = 8

^*^all occipital lesions were in the visual eloquence

The patients were categorized into two groups: eloquent group (EG) and non-eloquent group (NGE). The orbital and spinal cord cavernomas were excluded from the subgroup statistics for a reasonable comparison between the EG and NEG.

### Imaging protocols

Brain MRI was routinely performed within one week before surgery, mostly for neuronavigation purposes. A standard presurgical workup was made, including functional MRI, neurophysiology testing, and epilepsy work-up when needed. Postoperative MRI was done within the first 3 months, then yearly, or at the last follow-up. The imaging studies were read by a neuroradiologist (MK).

Postoperative bleeding was defined as an extension of the bleeding outside the resection bed in the postoperative imaging studies.

### Surgical strategy

The patients were operated on in our institute by different neurosurgeons. The aim of the surgery was a total lesionectomy. The treatment approach was harmonious in all cases. Different microsurgical approaches were selected depending on the case. A combination of intraoperative neuronavigation, ultrasound, awake surgery, or neurophysiologic monitoring was used as needed.

### Last follow-up

The patients were invited for a follow-up, so-called last follow-up, in which 44 patients of the present study population could attend, and they were distributed as follows: EG *n* = 27, NEG = 14, orbital *n* = 2, spinal cord *n* = 1. These patients were interviewed between 2017 and 2019.

In this follow-up, neurological status with an updated MRI was reviewed. The individuals were then asked to answer validated questionnaires of Quality of Life (QoL) of Short-Form 12 (SF12) [32], overall satisfaction, and reemployment status.

### Quality of life via Short-Form 12 (SF12)

The overall respondents’ rate of the SF12 questionnaire was 63.8% (44 out of 69). The mean interval between the surgery and the survey was 8.7 years.

SF12 survey contains 8 domains: (1) general health perceptions (GH general health); (2) limitations in usual role activities due to physical problems (RF functional role); (3) bodily pain(BP); (4) physical activities limitations due to health problems (PF physical functioning); (5) energy and fatigue (VT vitality); (6) limitations in social activities due to physical and emotional problems (SF social functioning); (7) limitations in usual role activities because of emotional problems (RE emotional role); and (8) general mental health, psychological distress and well-being (MH mental health).

The first 4 domains represent physical health and the last 4 represent mental health. The sum of each 4 domains generates a global score; physical composite scale (PCS) and mental health composite scale (MCS).

The sum of the questions in each of these 8 sections and the 2 global scores are transformed into a 0 to 100 scale. A lower score (0) represents more disability, whereas a higher score means less disability, and 100 points equivalent to no disability. Results from this test were re-calculated as described in SF-12 Health Survey Manual [32] and compared to normative data from the general German population [35].

The evaluation of these results focused on a comparison between (1) the two groups EG and NEG and (2) the study population, EG, and NEG with a normative German population, respectively [35].

### Statistical analysis

All data were expressed as mean and standard deviation. For subgroup analysis, Fisher’s exact test was used for categorical variables. An independent two-sample t-test was performed for continuous variables. Both were used for the presentation of quantitative differences among subgroups. Additionally multivariate ANOVA was done to assess the difference in subgroups in regards to outcome related to mRS using STATA 13 (StataCorp. 2013. Stata Statistical Software: Release 13. College Station, TX: StataCorp LP.). Statistical analyses for the QoL were performed using commercially available software (SPSS, Ver. 20.0, IBM Corp., Armonk, New York, USA). Two-tailed *P* value < 0.05 was considered significant, and *P* < 0.01 as highly significant.

## Results

### Demographic data and clinical presentation

Sixty-nine patients made up the study cohort, including 30 women (43.48%) and 39 men (56.52%). The average age at surgery was 41.3 years (SD 16.2, range 16–78 years). Table 3 demonstrates a summary of the demographic and clinical characteristics of the whole study population, EG, and NEG.

**Table 3:** Demographic and clinical characteristics of 69 patients with CM divided into two groups: eloquent group (EG) and non-eloquent group (NEG)

No. of patients	Study population 69	Eloquent group 41	Non-eloquent group 22	Significance
Mean age at the operation in years Female (%)	41.7 (SD 16.2) (range, 16–78 years) 30 (43.5%)	42.4 (SD 16.6) 22 (53.7%)	40.0 (SD 16.2) 7 (31.8%)	
Mean duration of presentation in months	40.2 (SD 80.9) (range, 1–400)	48.4 (SD 94,5)	26.3 (SD 63.2)	
Symptoms (%)				
- FND	− 29 (42.0%)	− 19 (46.3%)	− 4 (18.1%)	
- seizure	− 24 (34.9%)	− 13 (31.2%)	− 11 (50.0%)	
- FND and Seizure	− 8 (11.6%)	− 6 (14.6%)	− 2 (9.1%)	
- Incidental	− 4 (5.8%)	− 1 (2.4%)	− 3 (13.6%)	
- Headache	− 4 (5.8%)	− 2 (4.9%)	− 2 (9.1%)	
Mean follow-up in years	6.5 (SD 4.6) (range, 1–18)	5.8 (SD 4.0)	7.6 (SD 5.6)	
Mean CM size in mm	18.1 (SD 10.4) (range 4–56)	18.4 (SD 9.8)	16.1 (SD 9.0)	NS
Mean operation time in minutes	223.0 (SD 100.0) (range, 82–573)	240.2 (SD 100.1)	184.6 (SD 94.6)	*P*-Value = 0.0362 †
Mean length of stay in days	9.9 (SD 4.3) (range, 5–28)	10.2 (SD 4.9)	9.5 (SD 3.4)	NS
Zabramski class [37]				
I/II/III/IV	22/40/7/0	13/27/1/0	5/11/6/0	
Rate (%)	32/58/10/0	32/66/2	23/50/27/0	

*CM* cavernous malformation; *FND* focal neurological deficit; *NS* non-significant; *SD* standard deviation

Values represent numbers of cases (%) unless otherwise indicated. Mean values are presented with standard deviations. p values are for comparison of the difference between subgroups

Among these patients, 29 (42.0%) presented with FND, 24 (34.8%) with symptomatic focal epilepsy, and 8 patients (11.6%) with both FND and seizures.

Four patients (5.8%) presented only with long-standing nonspecific headaches, and in 4 (5.80%) patients the cavernoma was an incidental finding, where 2 of them presented because of remote bleeding and the other 2 due to non-cavernoma-related epilepsy.

The mean duration of preoperative clinical history was 40.2 months (SD 80.9) of all cavernomas. The mean last available follow-up was 6.5 years (SD 4.6).

### Genetic profiles

Since a positive family history and multiple CMs are indicative of the familial form of CCM, genetic counseling was offered to all study participants who met these criteria. Three of the eight patients gave their written informed consent for genetic analyses of the three disease-associated genes *CCM1* (also known as *KRIT1*), *CCM2*, and *CCM3* (also known as *PDCD10*). A pathogenic *CCM1* frameshift variant was identified in one patient, and a *CCM2* splice site mutation, which was also classified as pathogenic, according to the American College of Medical Genetics and Genomics (ACMG) guidelines [26], was detected in two brothers. [21]

### Imaging and surgical outcome

The most common type of MRI presentation was type 2 Zabramski grade (58.0%).

**Fig. 1:**
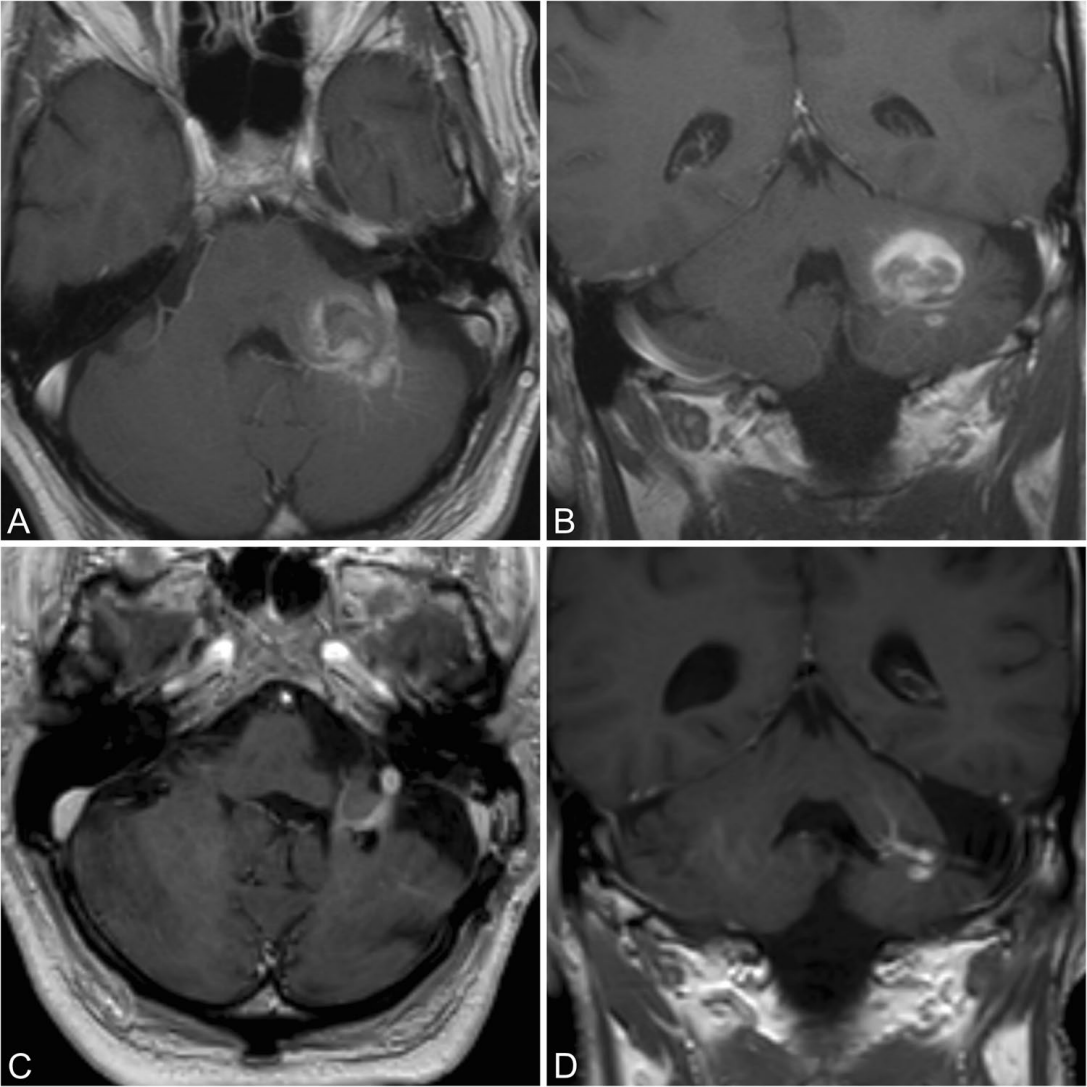
CM in the left middle cerebellar peduncle. Preoperative axial (**a**) and coronal (**b**) T1-weighted images with gadolinium demonstrating cavernoma with associated DVA. Postoperative axial (**c**) and coronal (**d**) T1-weighted images with gadolinium, showing complete resection of the CM with preservation of the DVA

Complete resection was achieved in 67 patients (97.1%) determined by intraoperative inspection and postoperative MR imaging. An accompanying DVA was detected in 19 patients (27.5%) and was preserved in all cases (Fig. 1). The duration of the operation in EG was longer than for the NEG; the difference was significant ( *P* = 0.0362, t-test).

### Complications and surgical morbidity

Directly postoperatively, new FND or worsening of the presenting symptoms, as noted in Table 4, was observed in 31.7% of the EG and 4.5% of the NEG. An immediate worsening of one or more grades on the mRS was seen in 19.5% of patients with eloquent cavernomas and 4.5% of patients with non-eloquent cavernomas corresponding to the surgical morbidity. There were no cases of mortality.

**Table 4:** List of the postoperative complications

	No	Comments:
FND		
-Paresis	5	Some patients had mixed FND
-Paresthesia	4	
-Dysphasia	1	
-Cognitive dysfunction	1	
-Ataxia	2	
-Diplopia	3	
-Visual field defect	1	
Postoperative bleeding#	4	
Medical problem		
Deep vein thrombosis	1	
Pulmonary embolism	1	PE occurred in a patient with brainstem cavernoma
Wound infection	1	1 reoperation
CSF Fistula	2	1 reoperation

^#^ bleeding that slightly extended from the resection bed in the post-operative imaging

*CSF* cerebrospinal fluids; *CRE* cavernoma related epilepsy; *FND* focal neurological deficit; *PE* pulmonary embolism

**Fig. 2:**
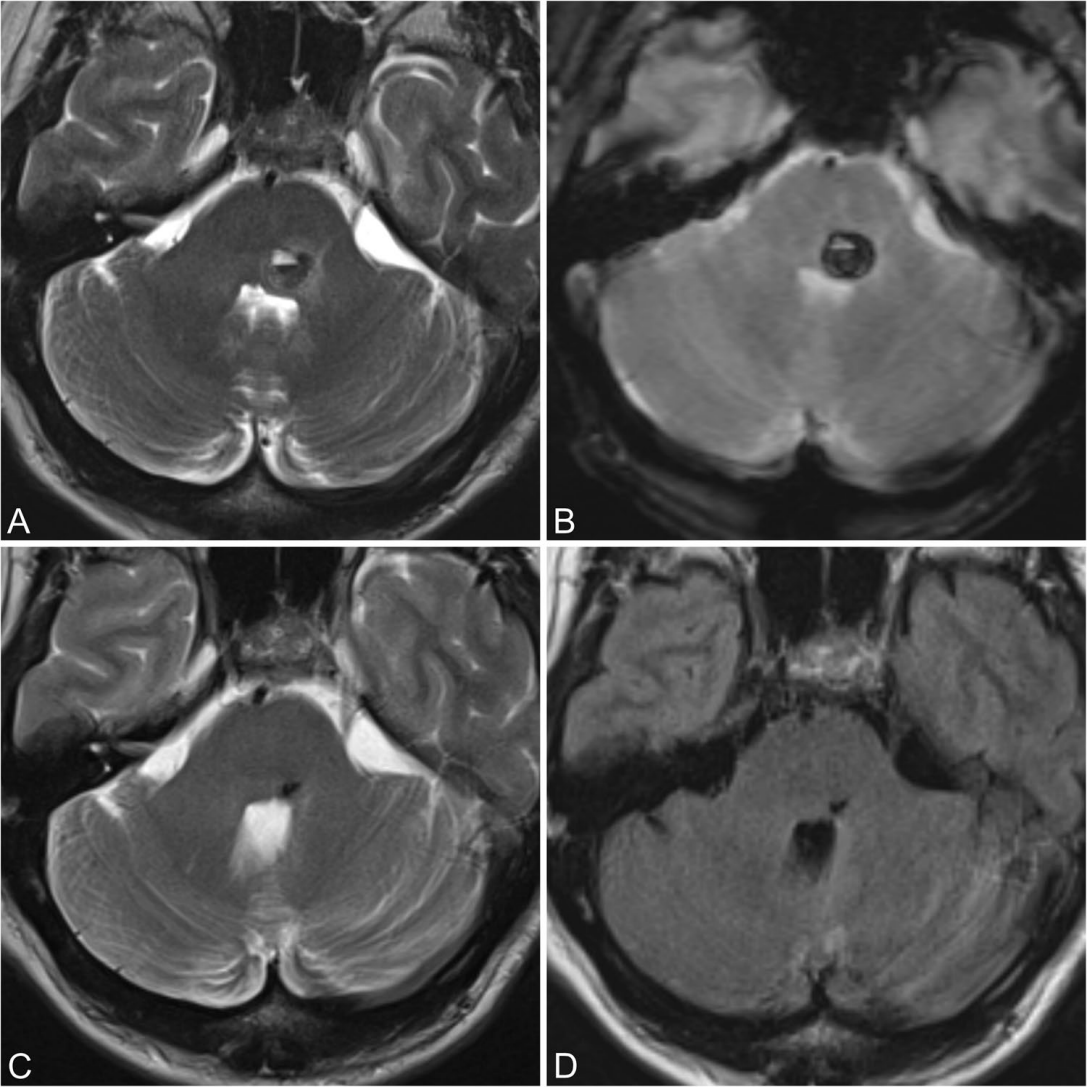
Pontine CM. Preoperative axial T2-weighted image (**a**) and axial gradient echo image (**b**). Postoperative axial T2-weighted image (**c**) and axial T1-weighted image (**d**). The patient presented with mixed neurological deficits with mRS 2 at the presentation that was better at the follow-up and reported an overall favorable quality of life

At the late follow-up, after a mean follow-up time of 6.5 years, the status was equal or better than the baseline at presentation in 85.4% of patients in the EG vs 100% in the NEG. (Fig. 2).

**Fig. 3:**
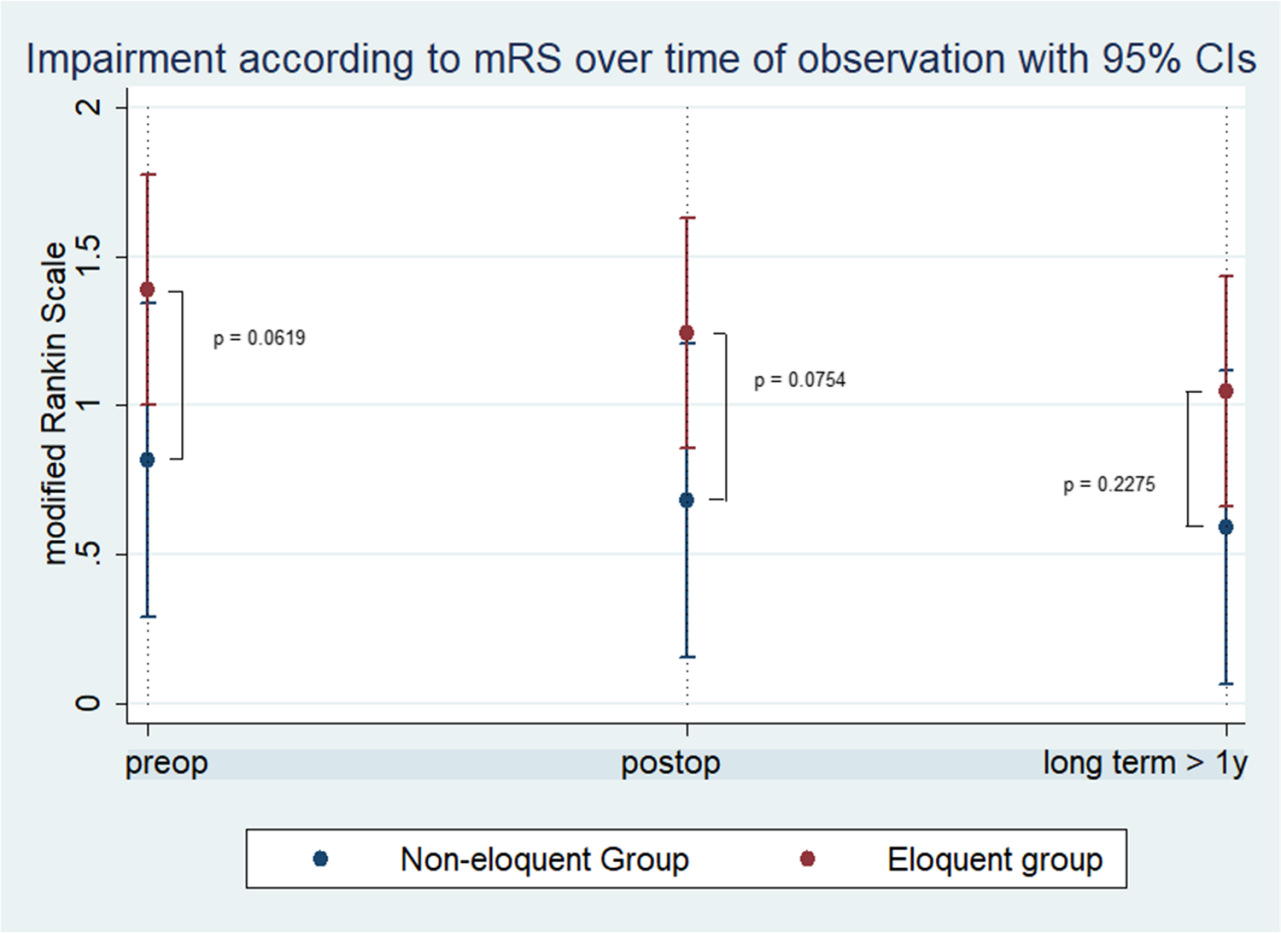
Difference between the eloquent group and non-eloquent group according to mRS over the course of the disease. The p values depicted in the diagram indicate the difference between both groups for each time point of evaluation. mRS = modified Rankin scale; preop = preoperative; postop = postoperative; long term = at the last follow-up 6.5 years ± 4.6 (range, 1–18)

An unfavorable outcome (mRS > 2) was seen in 12.2% of patients in EG and 0% in NEG at the last follow-up. The change of mRS throughout the course of the disease is illustrated in (Fig. 3). A statistically non-significant improvement of neurological deficit (mRS) was observed in both groups between the preoperative presentation and long-term follow-up; NEG improvement of − 0.11 on the mRS (*p* = 0.496) vs EG improvement of − 0.17 on the mRS (*p* = 0.265).

Twelve out of 63 patients (19.0%) who presented with intracranial CM had preoperatively chronic disabling headaches. At the last follow-up, only one patient (1.6%) had persisting headache episodes.

### Rebleeding and reoperation

Postoperative bleeding was detected in the routine postoperative brain image in 4 patients (5.7%) without any clinical manifestation. None of them required surgical evacuation.

As demonstrated in Table 4, two postoperative complications necessitated a reoperation, due to CSF fistula, and postoperative wound infection.

### Epilepsy outcome and antiepileptic drugs (AED)

Regarding cavernoma-related epilepsy, 23 out of 29 patients (79.3%) reported seizure-free or only rarely disabling seizures after surgery (Engel classification 1) [34], while 31.0% of the patients were able to discontinue the AED and 17.2% of patients could decrease at least one AED.

### Return to work and patient satisfaction

In response to the supplementary question at the last follow-up (*n* = 44) (“Did the operation meet your expectations about the postoperative course of the treatment”), 88.8% of the patients expressed their satisfaction with the treatment, while 11.2% found that the surgical treatment did not meet their expectations.

Regarding reemployment or return to baseline activity, 16 out of 27-asked patients (59.3%) in the EG were able to return to work compared to 12/14 patients (85.7%) in the NEG, including two retired patients that were able to do the housework as before the operation. This difference between the two groups was not significant.

### Health-related quality of life (QoL)

The assessment of QoL with the SF12 questionnaire was performed during the last follow-up. The overall respondents’ rate of the SF12 questionnaire was 44 cases (63.8%). The mean interval between the surgery and the survey was 8.7 years.

**Fig. 4:**
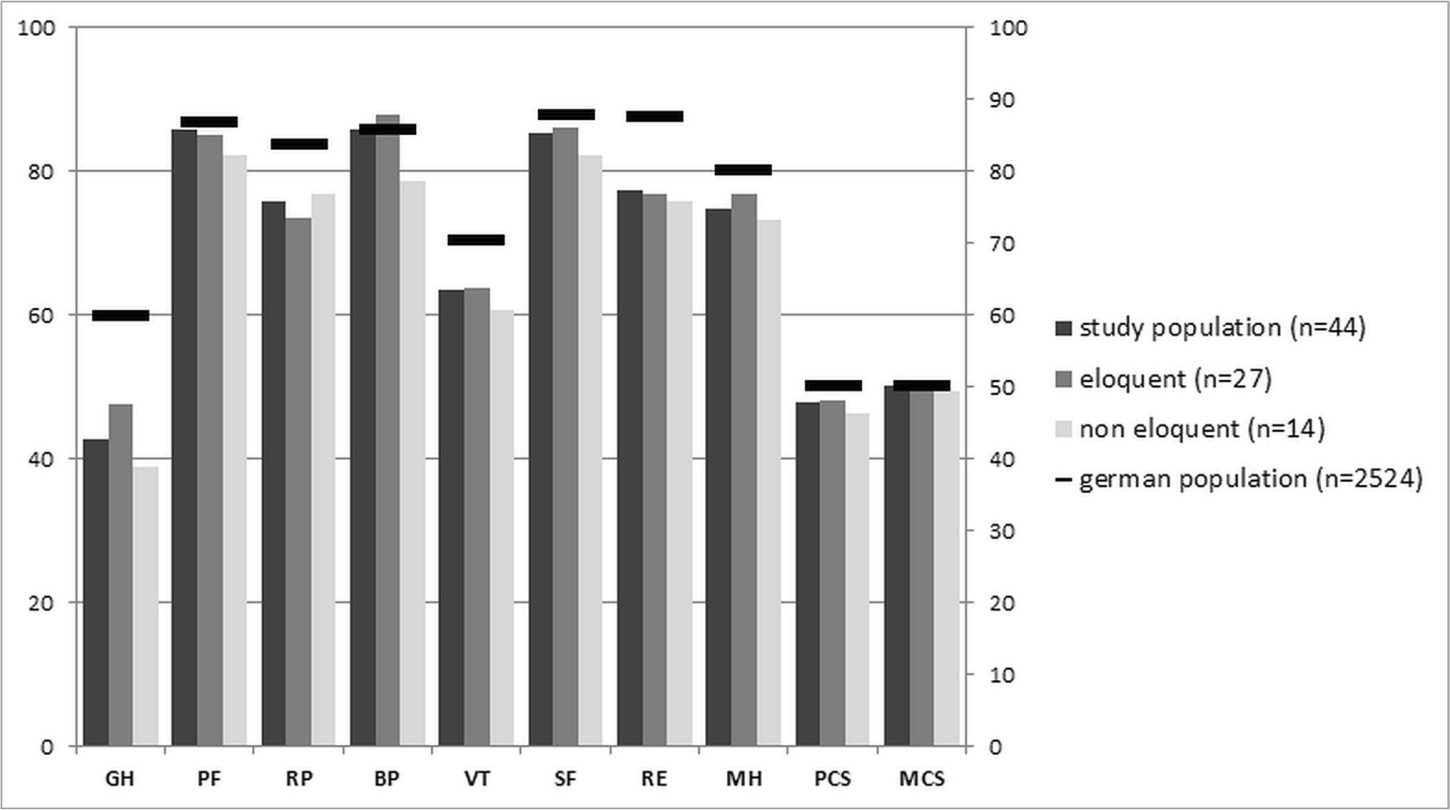
Barograph displaying the results of SF12 questionnaire for the study population (black), eloquent subgroup (gray), non-eloquent subgroup (white), and healthy German population (black line). The 8 items and the 2 global scores of the questionnaire are presented on the x-axis. The scale is from 0 to 100 demonstrated on the y-axis. The study population that made up the last follow-up (*n* = 44) was distributed as follows: eloquent group *n* = 27, non-eloquent group *n* = 14, orbital *n* = 2, spinal cord *n* = 1. BP = bodily pain; GH = general health perceptions; MCS = mental health composite scale; MH = mental health; PCS = physical composite scale; PF = physical functioning; RE = role emotional; RP = role physical; SF = social functioning; VT = vitality

The results are demonstrated in Table 5 and illustrated in Fig. 4.

1. QoL according to eloquence, EG vs. NEG: the subgroup analysis showed no statistical difference between both subgroups after a long follow-up. The EG showed a slightly better score in general health (GH) and bodily pain (BP) domains, where the physical role (RP) was more limited in the EG.
2. Comparison with the normative German population (norms): The physical and mental component score of the entire study population and the subgroups when compared with norms showed no statistically significant difference. The patients scored equal to the norms regarding the mental component score.

**Table 5:** Results of SF-12 questionnaire for study population, subgroups, and healthy population (Germany) [35]

	Study n44* Mean (SD)	German population *n* 2524 Mean (SD)	Study vs norms	EG *n* 27 Mean (SD)	NEG *n* 14 Mean (SD)	EG vs norms	NEG vs norm	EG vs NEG
GH	42.73 (27.05)	59.79 (23.10)	*P* < 0.0001 t-test	47.59 (26.36)	38.93 (27.89)	*P* = 0.0064 t-test	*P* = 0.0007 t-test	NS
PF	85.80 (27.70)	86.76 (24.32)	NS	85.19 (27.09)	82.14 (30.11)	NS	NS	NS
RP	75.85 (27.41)	83.61 (22.55)	*P* = 0.0243 t-test	73.61 (30.29)	76.79 (23.95)	*P* = 0.0225 T-Test	NS	NS
BP	85.80 (21.16)	85.66 (23.15)	NS	87.96 (17.50)	78.57 (27.49)	NS	NS	NS
VT	63.64 (22.53)	70.40 (18.56)	*P* = 0.0171 t-test	63.89 (25.32)	60.71 (18.90)	NS	NS	NS
SF	85.23 (23.08)	87.83 (20.10)	NS	86.11 (21.18)	82.14 (28.47)	NS	NS	NS
RE	77.27 (26.72)	87.51 (20.45)	*P* = 0.0011 t-test	76.85 (27.89)	75.89 (27.50)	*P* = 0.0073 t-test	*P* = 0.0344 t-test	NS
MH	74.72 (16.06)	80.10 (22.73)	NS	76.85 (17.92)	73.21 (11.87)	NS	NS	NS
PCS	47.81 (6.00)	50.00 (10.12)	NS	48.04 (5.97)	46.31 (5.97)	NS	NS	NS
MCS	50.11 (9.12)	49.99 (10.08)	NS	50.69 (9.61)	49.43 (9.12)	NS	NS	NS

^*^n = 44 divided in EG *n* = 27, NEG = 14, orbital *n* = 2, and spinal cord *n* = 1

*BP* bodily pain; *EG* eloquent group; *GH* general health perceptions; *MCS* mental health composite scale; *MH* mental health; *NEG* non-eloquent group; *NS* not significant; *PCS* physical composite scale; *PF* physical functioning; *RE* role emotional; *RP* role physical; *SF* social functioning; *VT* vitality

Regarding physical health analysis; the general health (GH) was significantly better in norms compared to subgroups.

The physical role (RP) also showed a better score in norms; this difference was significant compared to EG and the study population (*p* < 0.05, t-test), but not to the NEG.

In mental health analysis; the patients reported vitality (VT, energy level) and emotional role (RE) perception significantly inferior to the normative population as Table 5 illustrated.

## Discussion

### Neurosurgical outcome

In this study, 94.1% of patients were symptomatic, 88.4% of the patients presented with new neurological symptoms before surgery. Short-term morbidity after surgery was seen in 19.5% of cases in the EG versus 4.5% in the NEG. Long-term morbidity was in 14.6% of cases in the EG versus 0% in the NEG.

The results in the presented study were comparable to the outcome stated in the literature. A larger study (*n* = 79) reported 97.4% of the patients had better or identical status after supratentorial cavernoma resection in eloquent areas at last follow-up [6]. Wostrack et al. (*n* = 41) reported 47% of patients had a new postoperative deficit. At follow-up, 80% recovered to at least preoperative status [36]. In a recent study by Sanmillan et al. that assessed the surgical outcome of 20 patients who presented with cavernoma in eloquent location, 50% of patients had transient deterioration that all recovered after 1 year [28].

Regarding brainstem cavernomas, 62.5% of our patients had a better or identical neurological outcome at the last follow-up. A study of a larger cohort of brainstem cavernomas (*n* = 260) showed a rate of new postoperative FND in 53% of the patients and permanent morbidity with new deficits remained in 36% of them after a mean follow-up of 51 months [1].

When left untreated, the eloquent lesions and especially brainstem lesions will deteriorate due to the increased rebleeding rate (estimated 6.3% patient/year for non-brainstem and 32.2% patient/year for brainstem lesions that could also reach up to 52.7% patient/year) [31]. Knowing the natural history of eloquent cavernomas and to a lesser extent brainstem cavernomas justifies the decision of surgery in these eloquent areas [18, 27, 36].

The fear of surgical morbidity should be reconsidered when we look at the prolonged follow-up of the patients, where the neurological deficits will mostly recover and the risk of hemorrhage from the cavernoma will decrease or even be eliminated by the complete resection.

### Return to work

The rate of patients returning to work in EG was lower than the NEG (59.3% vs 85.7%). Correlated to QoL and mRS results, the limiting physical role (RP) and unfavorable outcome (mRS > 2) at the last follow-up (12.2% vs 0% as shown in Table 1) could contribute to the difference between the two groups. Other factors such as age, gender, and AED status did not show a correlation to this difference.

Our results from patients in the EG returned to work compared to a larger multicentric survey from Zanello et al. showed a lower proportion of patients returning to work (59.3% vs 88.6%, respectively) [38]. This difference between our results and this survey is hard to explain since both studies lack a suppurative socioeconomic data of these patients. The mean age of both populations (42.4 vs. 40.2 years) as well as the slight female predominance (53.7% vs 59.6%) and functional worsening at the follow-up (14.6% vs 12.9%) were similar.

### Health-related quality of life

The QoL of patients after cavernoma resection was assessed in four previous publications, including three studies for brainstem cavernomas only. Cornelius et al. compared the quality of life between the brainstem and non-brainstem cavernomas. They found, expectedly, that patients with a brainstem lesion were far more inferior regarding physical health domains of the QoL. Previous studies about QoL of patients after brainstem cavernoma surgery reported a favorable QoL in terms of mental health aspects [7, 9, 17].

Another study has been conducted considering patients’ satisfaction with cavernoma-related epilepsy. The assessment, however, took part through a survey per mail [33].

The difference in this study is seen in the physical functioning (PF) domain. Our patients reported a good score in this domain, in contrast to previous studies. Our explanation is as follows: the previous studies included brainstem cavernomas as a group and compared it with the normative population [7–9, 17], or with non-brainstem group [7]. Unsurprisingly, the brainstem group reported an inferior score regarding physical functioning. In the present study, patients in the eloquent group could have a visual field defect or mild hemiparesthesia and still report a good physical functioning perception compared to the brainstem group; this could explain the observed difference.

Furthermore, none of the studies mentioned above assessed the QoL of their study population sorted by functional location of the lesion (eloquence). They instead compared brainstem with non-brainstem cavernoma or normal population. The non-brainstem cavernomas have a wide range of presentations from asymptomatic to significant disability depending on the eloquence. The perception of QoL in patients with disabilities differs from patients with oligosymptomatic state. Moreover, some recommendations from the Angioma Alliance for cavernoma management from Akers et al. relied on the eloquence of the lesion [3]. Hence, the present study assesses the QoL distributed accordingly.

To summarize the results about the QoL, we found that patients with cavernoma located in eloquent regions after surgery mostly had a non-inferior QoL compared to NEG except the physical role (RP) domain. Compared to their normative correspondence, the EG reported general health (GH) perception inferior to norms, which was related to limited physical and emotional roles.

Our results in line with the results of the previous studies show that the QoL accompanied by the neurological outcome is an essential and powerful element to support the decision for treating CNS cavernomas, especially if the cavernoma is in an eloquent area.

### Strength and limitation of the study

A major strength of this study is that we conducted face-to-face interviews for QoL measurements in more than half of the patients in a prolonged period after surgery (mean time-to-interview 8.7 years). Some previous studies conducted such interviews via mail or telephone only [7, 9, 33]. Moreover, the correlation to the normal German population was from the same survey done in 2018; that time is comparable to the time of the follow-up of this study.

However, there are some limitations to our study. First, we only recruited participants from a single tertiary care center, and the results might not be generalized to other settings or populations. Second, this study is a retrospective study that lacks a randomized controlled group. Third, the sample size for the QoL assessment (*n* = 44) with a further subdivision of EG (*n* = 27) and NEG (*n* = 14) is relatively small. This could lead to poor statistical significance when testing the QoL domains between the two groups, EG and NEG.

Fourth, as the previously conducted studies about QoL of cavernoma patients, there was no preoperative QoL assessment or socioeconomic data of our patients to correlate the improvement of QoL postoperatively due to the retrospective design of the study.

In summary, the present study adds information to the literature about QoL after cavernoma surgery, which is needed to validate patients’ well-being after surgical treatment. The comparison between eloquent and non-eloquent cavernomas, created interesting and new results after prolonged follow-up. The assessment of CNS Cavernoma QoL has only rarely been performed [7–9, 17].

## Conclusion

At a late follow-up, the surgical morbidity was transient in the NEG and mostly recovered in the EG (85.4% of patients). Regarding QoL, patients after eloquent cavernoma resection reported a non-inferior QoL in most SF12 domains (except for physical role RP) compared to NEG. However, they reported general health perception inferior to norms, which was affected by the limited physical and emotional roles. These results of how these patients are doing years after surgery could improve the decision-making as well as the patient counseling for future encountered cases of cavernomas in eloquent and non-eloquent regions. When reporting the neurosurgical outcome after CNS cavernoma resection, the QoL outcome is an essential measurement. For future studies, preoperative QoL measurements for the assessment of QoL dynamics are highly recommended.

## Data Availability

Raw data were generated at University Medicine Greifswald. Derived data supporting the findings of this study are available from the corresponding author LS on request.
